# Supplementary material for: Possible Association between Genetic Diversity of Hepatitis B Virus and Its Effect on the Detection Rate of Hepatitis B Virus DNA in the Placenta and Fetus
Source: Viruses. 2023 Aug 12;15(8):1729. doi: 10.3390/v15081729 (PMC10458115; doi:10.3390/v15081729)
Supplement: Supplementary file 1 [file viruses-15-01729-s001.zip › viruses-2521501-supplementary.pdf]

Table S1: List of primers used for amplification of S and C HBV genome in this study

Target HBV gene	Nucleotide	Primer's sequence (5'-3')	PCR product size (bp)	Reference
S-gene	2819-842	F- TCACCATATTCTTGGGAAC<br>R- AGGGTTTAAATGTATACCCA	1206	(30)
C-gene	1552-2476	F- TCTGTGCCTTCTCATCTG<br>R- CCCACCTTATGAGTCCAAGG	924	(31)
	1584-2396	F- ACTTCGMBTCACCTCTGCACG T<br>R- GTCKGCGAGGYGAGGGAGTT	748	(32)

30 Sagnelli C, Ciccozzi M, Pisaturo M, Zehender G, Lo Presti A, Alessio L, et al. Molecular epidemiology of hepatitis B virus genotypes circulating in acute hepatitis B patients in the Campania region. J Med Virol. 2014;86(10):1683-93.

31 Tangkijvanich P, Sa-Nguanmoo P, Avihingsanon A, Ruxrungtham K, Poovorawan K, Poovorawan Y. Characterization of hepatitis B virus mutations in untreated patients co-infected with HIV and HBV based on complete genome sequencing. J Med Virol. 2013;85(1):16-25.

32 Chook JB, Teo WL, Ngeow YF, Tee KK, Ng KP, Mohamed R. Universal Primers for Detection and Sequencing of Hepatitis B Virus Genomes across Genotypes A to G. J Clin Microbiol. 2015;53(6):1831-5.
